# Effectiveness of the User-Centered “Healthcare CEO” App for Patients With Type 1 Diabetes Transitioning From Adolescence to Early Adulthood: Protocol for a Randomized Controlled Trial

**DOI:** 10.2196/59871

**Published:** 2025-01-13

**Authors:** Yueh-Tao Chiang, Hsing-Yi Yu, Pei-Kwei Tsay, Chi-Wen Chen, Chi-Wen Chang, Chien-Lung Hsu, Fu-Sung Lo, Philip Moons

**Affiliations:** 1 School of Nursing, College of Medicine Chang-Gung University Taoyuan Taiwan; 2 Division of Pediatric Endocrinology & Genetics, Department of Pediatrics Chang-Gung Memorial Hospital Taoyuan Taiwan; 3 Department of Nursing New Taipei Municipal Tu-Cheng Hospital New Taipei Taiwan; 4 Department of Public Health and Center of Biostatistics, College of Medicine Chang-Gung University Taoyuan Taiwan; 5 College of Nursing National Yang Ming Chiao Tung University Taipei, Taiwan; 6 Department of Information Management Chang-Gung University Taoyuan Taiwan; 7 Graduate Institute of Management Chang Gung University Taoyuan Taiwan; 8 Master of Science Degree Program in Innovation for Smart Medicine Chang Gung University Taoyuan Taiwan; 9 College of Medicine Chang-Gung University Taoyuan Taiwan; 10 Department of Public Health and Primary Care KU Leuven-University of Leuven Leuven Belgium; 11 Institute of Health and Care Sciences University of Gothenburg Gothenburg Sweden; 12 Department of Paediatrics and Child Health University of Cape Town Cape Town South Africa

**Keywords:** type 1 diabetes, transition, mobile health, treatment fidelity, diabetes, user-centered, adolescence, teenager, app, adolescent patients, early adulthood

## Abstract

**Background:**

Young patients aged 16 to 25 years with type 1 diabetes (T1D) often encounter challenges related to deteriorating disease control and accelerated complications. Mobile apps have shown promise in enhancing self-care among youth with diabetes. However, inconsistent findings suggest that further evidence is necessary to confirm the effectiveness of app-based interventions.

**Objective:**

This study aims to evaluate the effectiveness of the Healthcare CEO app in patients with T1D transitioning from adolescence to early adulthood.

**Methods:**

A 2 arms, double-blind, randomized controlled trial will be conducted over a 9-month period, with strategies designed to enhance treatment fidelity. The study expects to enroll 96 patients with T1D, aged 16 to 25 years. Participants will be randomly assigned to either the experimental or control group through central randomization. The intervention will be implemented using the Healthcare CEO app, which consists of 11 interfaces. The research will compare differences in disease control outcomes, confidence in self-management, self-care behaviors, emotional distress, quality of life, and specific diabetes-related knowledge between the 2 groups at baseline and 3, 6, and 9 months after intervention. Additionally, changes within the experimental group will be analyzed before and after the intervention.

**Results:**

The study was funded in August 2020. It was originally scheduled from August 2020 to July 2022 but was interrupted by the COVID-19 pandemic after enrolling 38 participants, with preliminary results anticipated for publication by November 2024. Recruitment resumed in August 2023, with findings expected to be finalized by July 2025.

**Conclusions:**

The Healthcare CEO app is a comprehensive solution tailored specifically for individuals with T1D transitioning from adolescence to early adulthood. This innovative app has the potential to improve the quality of care for adolescents with T1D during this critical stage and may serve as valuable evidence in support of app-based intervention strategies.

**Trial Registration:**

ClinicalTrials.gov NCT05022875; https://www.clinicaltrials.gov/study/NCT05022875

**International Registered Report Identifier (IRRID):**

DERR1-10.2196/59871

## Introduction

### Overview

Type 1 diabetes (T1D) is the most common type of diabetes among youth younger than 18 years [1]. The incidence of T1D has exhibited a decline in the 0-9 years age group and an increasing trend in the 10-14 years age group [2,3]. Notably, T1D accounts for approximately 0.59% of all diabetes cases in Taiwan, and its incidence in patients aged 19 years or younger is 5.17 out of 100,000 while its prevalence is 0.05%. The prevalence and incidence of T1D in patients aged 19 years or younger increased significantly from 2005 to 2014 [4]. With this increase, the demand for care during the transition phase between adolescence and early adulthood is also expected to increase [5]. Therefore, understanding the life experiences and health care needs of adolescents with T1D in this transition phase and comprehending the customization of interventions to meet these needs is crucial in T1D care.

### Background

#### Encounters and Challenges of Patients With T1D in the Transition Phase From Adolescence to Early Adulthood

A transition phase is when an individual encounters significant life events or environmental changes. It is fraught with uncertainty and requires adaptation by learning new skills and strategies [6]. In nursing, the antecedents of the transition process are transition events that can be classified into 4 categories: developmental, situational, health‐illness, and organizational transitions [6]. Moreover, providing nursing support to patients in transition may contribute to role mastery and maintenance of physical and emotional well-being [7].

The encounters and challenges of patients with T1D transitioning from adolescence to early adulthood are wide-ranging and possess unique characteristics. The developmental transition from adolescence to early adulthood is often characterized by a rapid increase in height and body weight, as well as dramatic hormonal changes. Therefore, individuals with T1D in this transition commonly face the physiological challenge of maintaining stable blood glucose levels [5,8] and are at a high risk of poor disease control [9,10]. A study of individuals registered in the T1D Exchange Clinic Registry (a large-scale registry of patients with T1D in the United States) revealed that the mean hemoglobin A_1c_ (HbA_1c_) level in 18- to 25-year-olds was 8.7% (SD 1.9%) [11], which is higher than the average. The inability to achieve optimum blood glucose control is one of the greatest stressors faced by patients with T1D and may even cause anticipatory nervousness, which in turn affects sleep quality [12]. Other key stressors include the quest for independence, the burden of disease-related self-care, and the implementation of disease self-management [13,14]. Challenges faced by young patients with T1D in situational transition include the deconstruction of peer relationships caused by education system transition, establishment of sexual relationships, worries regarding the health and genetic inheritance of offspring, and reconstruction of physician-patient relationships due to workplace changes or home relocation [13,15,16]. During health-illness transition, especially from late adolescence to early adulthood, patients often become hospitalized owing to acute complications such as ketoacidosis and hypoglycemia. The need for frequent blood glucose monitoring and the transition from hospitalization to discharge often causes disruptions to life routines [5,16,17]. Young patients with T1D often experience an organizational transition, from family-centered care to patient-centered care, at 16-25 years [18,19]. Difficulties faced during this transition period often include the lack of a comprehensive transition-phase care plan that meets patient requirements, negative health care–seeking experiences in adult care systems, termination of health insurance, and loss to follow-up [10,20,21]. In Taiwan, the pediatric care model is adopted for most patients with T1D transitioning from adolescence to early adulthood. Many patients consider the transition from pediatric to adult care unnecessary owing to trust in physician-patient relationships and the familiarity of medical environments. Moreover, approximately 25% of patients reported that physicians should engage in active discussions with patients and provide assistance for referrals to adult care [5].

#### Needs and Effectiveness of App-Based Interventions Among Patients With T1D in the Transition Phase From Adolescence to Early Adulthood

Support from health care providers and primary caregivers is crucial for patients with T1D in the transition phase [14,22]. Patients with T1D, their primary caregivers, and health care providers agree that the provision of technology-based care and the development of specific apps for T1D self-management are important health care needs that should be addressed [23,24]. Studies have shown that diabetes-related knowledge, diabetes-specific and general life stress, and emotional disorders can affect self-care behaviors, disease management effectiveness, and HbA_1c_ control among patients in the transition phase [25-28], with higher severity of emotional distress associated with poorer HbA_1c_ control and quality of life (QoL) [29,30]. Therefore, app-based intervention measures should aim to address these factors.

Among the studies on the use of app-based interventions for youth with T1D or those in the transition phase, Goyal et al [31] and Veazie et al [32] reported that app-based interventions improved HbA_1c_ levels. Additionally, Frøisland et al [33] found that app-based interventions resulted in a better understanding of disease-related knowledge, whereas app usage seemed to positively affect diabetes self-care. However, some longitudinal studies have revealed that app interventions had no significant effects on HbA_1c_ levels [34,35], frequency of hypoglycemic events [31], diabetes-related QoL [31,34], self-perceived diabetes management ability, and diabetes-related emotional distress [35]. Although young patients with T1D generally provide positive ratings for the usability and feasibility of user-centered apps [35,36], these inconsistent results indicate that further evidence is necessary to support the effectiveness of app-based interventions.

#### Knowledge Gaps

Currently, the available apps for diabetes management can be broadly classified into 5 categories: nutrition, physical activity, blood glucose monitoring, insulin titration, and insulin injection. Most apps merely possess a single function, although there is an increasing trend toward integration [37]. Additionally, the majority of Chinese-language apps provide general diabetes knowledge, which may not be applicable to adolescents or young adults, and do not cater to the specific needs of patients with T1D in the transition phase [5]. Furthermore, the effectiveness of such apps should be validated by long-term empirical evidence [24,38], and few studies have mentioned the adoption of strategies to enhance treatment fidelity [31,35]. Health apps developed for intervention should incorporate evidence-based content while fulfilling the needs and protecting the privacy of the target age groups to enhance effective disease self-management [39]. To address the specific needs of patients in the transition phase, we developed the Healthcare CEO app using a user-centered approach. This app targets challenges such as poor glycemic control; pressures related to self-care; effective disease self-management; and interpersonal issues, including the establishment of romantic relationships, concerns about the health and heredity of future generations, workplace adaptation, parent-child conflicts, and enhancing QoL. This study aims to present a comprehensive protocol for evaluating the efficacy of the Healthcare CEO app. This will involve a series of detailed analyses, including (1) comparing disease control outcomes, confidence in self-management, self-care behaviors, emotional distress, and QoL between the experimental and control groups, and (2) a comparison of differences in preintervention and postintervention disease control outcomes, confidence in self-management, self-care behaviors, emotional distress, and QoL in the experimental group.

#### Conceptual Framework of the Study

**Figure 1:**
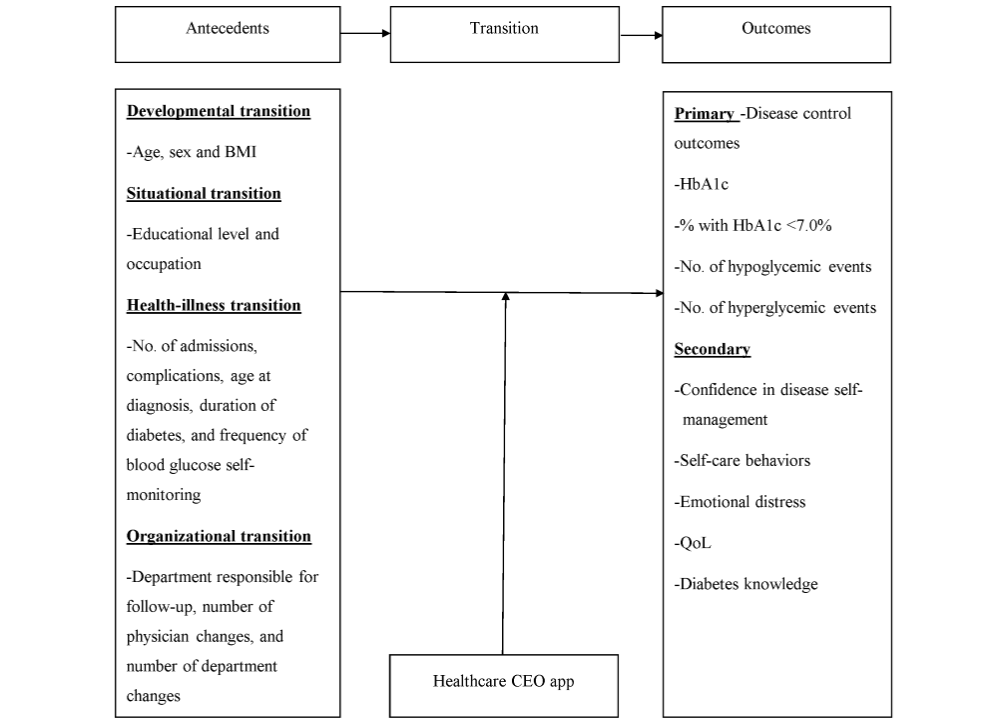
Conceptual framework of the study. HbA_1c_: hemoglobin A_1c_; QoL: quality of life.

Based on the literature described above, the transition phase theory was used as the framework of this study [6,40]. According to the transition phase theory, antecedents that trigger transition can be classified into 4 categories: developmental, situational, health illness, and organizational transitions. The Healthcare CEO app will be used as an intervention measure for patients with T1D transitioning from adolescence to early adulthood, and the effectiveness of the intervention in improving disease control outcomes, confidence in disease self-management, self-care behaviors, emotional distress, and QoL will be investigated, as shown in Figure 1.

## Methods

### Design

A 9-month, 2-arm, parallel-group, double-blind, randomized controlled trial will be conducted. Participants will be randomly assigned to the experimental or control groups, and treatment fidelity monitoring strategies will be adopted to enhance the consistency and fidelity of intervention measures.

### Participants

#### Inclusion and Exclusion Criteria

Participants will be enrolled from the outpatient clinic and wards of the Department of Pediatric Endocrinology and Metabolism, at a medical center in Northern Taiwan. The inclusion criteria are as follows: (1) a confirmed diagnosis of T1D from an endocrinologist before the age of 16 years and a disease duration of >6 months; (2) aged 16-25 years; (3) mean HbA_1c_ level ≥7.5% one year before inclusion; (4) ability to communicate in Chinese or Mandarin; (5) owning a smartphone with internet access; (6) agreeing to voice recording while explaining the treatment process; and (7) signing the informed consent sheet prior to participation—for minors, a legal representative must provide consent and sign the informed consent sheet. Patients with T1D with concomitant metabolic diseases, chromosomal aberrations, major illnesses, and cognitive impairment will be excluded from the study because their health care needs during the transition phase may differ from those who experience T1D only.

#### Sample Size

The required sample size was estimated using the G*Power program (Heinrich-Heine-Universität Düsseldorf) for repeated measures with 2 groups, 9 measured variables, power of 0.80, α set at .05, an estimated Cohen effect size of 0.25, and a correlation coefficient of 0.5. The sample size for each group was calculated as 36. By allowing for a potential 25% dropout rate, as suggested by Goyal et al [31], a final sample size of 96 participants (48 participants in each group) was established.

#### Sample Accessibility

The cumulative number of patients in the transition phase, aged 16-25 years, at the study site is expected to exceed 300. For each enrolled participant, the study explanation and data collection process is expected to last approximately 1 hour. Based on our research team’s qualitative research experience, the on-site interview length for each participant was 1-1.5 hour, whereas the refusal rate is expected to be approximately 10%. Therefore, each week, approximately 8 patients who fulfill the inclusion criteria will participate in the study. Thus, the number of valid samples expected each month is roughly 28-32.

### Randomization and Blinding

Randomization of the participants will be performed by a statistician, using the central randomization method in SPSS (IBM Corp). During participant enrollment, participants will be randomly assigned to the experimental or control groups in accordance with the instructions provided on the phone by the statistician; the participants and data collectors will be blinded to allocation. Blinding strategies include the following. First, the statistician will not be allowed to disclose the randomization results to others. Second, once a physician referral is received, the participant will be promptly taken to a private room. The data collection assistant will complete their duties and leave the room, and then 2 research assistants will come in and take over to administer the intervention phase. Third, participants of both the experimental and control groups will download the app, but the app content will differ. Fourth, to avoid differential demand characteristics, research assistants will emphasize to all participants that app usage is potentially beneficial for disease management.

### Description of the Intervention

#### Overview

**Figure 2:**
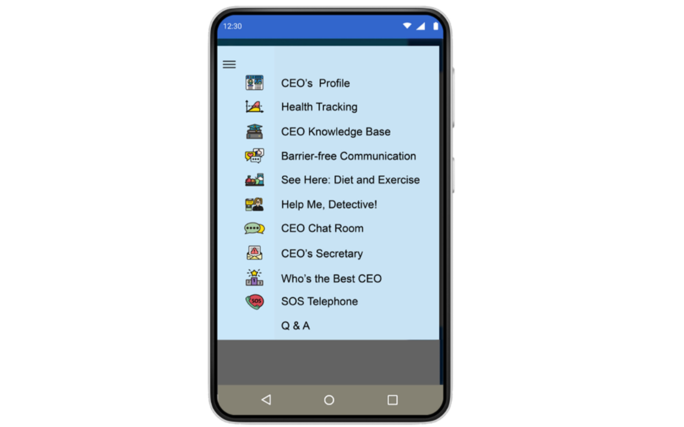
Interfaces of the Healthcare CEO app. CEO: chief executive officer.

The Healthcare CEO app was developed guided by the user-centered information systems research framework and comprised 3 key cycles: Relevance, Rigor, and Design [41]. It was named after the self-expectations of patients in diabetes management, as patients with T1D often wished to become the “Chief Executive Officers” of their disease. The development and testing process of the Healthcare CEO app is detailed in published articles [42]. The app content was designed based on the results of previous qualitative and Delphi studies [5,23] and consisted of 11 interfaces (as shown in Figure 2): CEO’s Profile; Health Tracking; CEO Knowledge Base; Barrier-free Communication; See Here: Diet and Exercise; Help Me, Detective!; CEO Chat Room; CEO’s Secretary; Who’s the Best CEO; SOS Calls; and Q&A. The evidence-based content aims to enhance disease knowledge among individuals in the transitional phase, improve self-care behaviors, and bolster confidence in disease self-management. These were combined with interpersonal communication skills training, to alleviate disease-related emotional distress, ultimately improving disease control outcomes and QoL. The content, objectives, and expected outcomes of each interface are detailed in Table 1.

**Table 1:** Content of Healthcare CEO app.

Interface	Description of content	Purposes and expected outcomes
CEO’s Profile	Personal and disease data, including the following: name, nickname, date of birth, occupation, duration of disease, hospital, and attending physician. The patient can select the data to be openly displayed.	Establish basic data Review and confirm basic disease information through data entry
Health Tracking	Upload or enter data on blood glucose level, blood pressure, body weight, and insulin dosage. HbA_1c_^a^ targets can be set, and changes in HbA_1c_ can be analyzed and displayed as trends.	The status of HbA_1c_ control can be checked to enhance self-care motivation and confidence in disease self-management
CEO Knowledge Base	Basic and advanced knowledge of interest to patients with T1D^b^ in the transition phase is provided in the e-book format. Examples of basic knowledge include the following: impact of secondary sex characteristic development on blood glucose; blood glucose control targets in T1D; and tips for dieting, exercise, and travel during puberty. Examples of advanced knowledge are as follows: matters related to sex, genetics, gestation, and employment among patients with T1D. Both a pretest and posttest must be completed to determine the learning effectiveness of each article before the next article can be accessed.	Patients can enhance T1D-related knowledge on a need basis Patients can boost their confidence in disease management by accumulating disease-related knowledge. This will enhance self-care behaviors, disease control outcomes, and disease-related QoLc
Barrier-free Communication	Guidance for techniques in parent-child, peer, and workplace interactions is provided through video and e-book links. Questions and scenarios are designed to prompt patients to reflect on their communication methods and think about/enact their strategies for effective interaction.	Improve communication skills, enhance ability to deal with interpersonal difficulties, and reduce emotional distress
See Here: Diet and Exercise	Carbohydrate and calorie calculators and a food swap guide are provided. The calorie contents of preferred foods and calories burned in preferred exercises of patients in the transition phase are displayed in images. Patients can keep records using the handy checkbox interface or uploading photos.	Assist in disease management and self-care, which will in turn improve disease control outcomes and disease-related QoL
Help Me, Detective!	When the blood glucose level is not within the expected range or emergency complications occur, this interface allows for the recording of the time of event occurrence, uploading of blood glucose readings, photographs of the patient’s diet, exercise, or patient condition during the event, and documentation of the treatment measures adopted. These data can serve as a reference for discussion with the physician during outpatient visits, and physician advice can be input into the interface to guide the handling of future emergencies.	By recording and analyzing the causes of acute complications, the patient may devise improvement measures to enhance disease control, which will gradually enhance confidence in disease self-management and improve disease control outcomes
CEO Chat Room	An optional second password can be set to achieve 2 levels of privacy protection, which may be very important to patients in the transition phase. Two chat rooms named the “insider chat room” and “outsider chat room” have been designed. The insider chat room is reserved for patients using the app, with 2 themed chats organized monthly. Patients may invite individuals whom they trust (eg, attending physicians, close friends, or primary caregivers) and can engage in heart-to-heart talk using the outsider chat room, to develop their interpersonal networks and exchange views and ideas.	Provide interpersonal support and enhance disease-related knowledge and management strategies through experience sharing, which will alleviate emotional distress and strengthen disease self-management ability
CEO’s Secretary	This consists of 2 functions, namely “Reminders” and “Online consultations.” (1) Reminders: When trends of exceedances related to blood glucose occur, such as excessively high postdinner blood glucose level for 3 consecutive days, noninput of health data for ≥3 days, fewer blood glucose measurements than the required number of measurements for 3 days, and on the day before a scheduled outpatient visit, patients and app administrators will receive notifications and text message reminding them to perform relevant tracking actions. Reminders can also be set based on the needs of the patients; (2) Online consultations: The timing and theme of online consultation sessions can be booked in advance. Online consultations involve the clarification of doubts related to disease care through interactions with health care professionals.	Assist in disease management and provide individualized knowledge to enhance disease-related knowledge and confidence in self-care, thereby alleviating emotional distress and improving disease-related QoL Conditions for reminders can be set to reduce annoying reminders, to prevent resentment of app use
Who’s the Best CEO	One point is awarded every time the user performs data input on time, reads an article, devises a communication technique, and correctly enters data into the Help Me, Detective! interface, or enquires about online consultation. Ten points are awarded when the target HbA_1c_ level is reached. The patients with the highest number of points are listed weekly. At the end of each month in our study, the top 3 patients with the highest number of points will each receive an NT $300 (approximately US $9.24) voucher. Moreover, at the end of the study, the top 3 patients with the highest number of points will receive an NT $1000 (approximately US $30.79) voucher.	Enhance motivation and effort in self-care
SOS calls	Patients can set up 3 contact numbers to call for help quickly when necessary.	Assist in disease management and self-care, thereby improving disease control outcomes
Q&A	Patients who may be unaccustomed to asking questions in the chat room can have their queries answered via the provided email account.	Assist in disease management and self-care to improve outcomes

^a^HbA_1c_: hemoglobin A_1c_.

^b^T1D: type 1 diabetes.

^c^QoL: quality of life.

#### Experimental App Intervention

The complete Healthcare CEO app will be used to intervene in the experimental group. Two trained research assistants will supervise each other to guide the participants in the following aspects, according to the standard treatment manual: (1) account setup; (2) viewing of operation guidance videos; (3) demonstration of all app functions, getting participants to perform app operations, and clarifying doubts about app usage; (4) explaining troubleshooting methods; (5) providing contact information of app administrators; and (6) setting a HbA_1c_ control target. Between every 2 rounds of data collection, the research assistants will contact the participants to check if they have encountered issues or difficulties when using the app, although they will not exchange disease care-related information. After the completion of data collection, the app will be remotely uninstalled by the administrators at the backend.

#### Control App Intervention

Participants in the control group will only install the “CEO’s Profile” and “Health Tracking” interfaces of the Healthcare CEO app. In addition to explanations for the account setup and the use of the 2 interfaces, the research assistants will not provide any app-related information to the control participants. The status and difficulties of app use will also not be tracked. After the last round of data collection, the full version of the Healthcare CEO app will be provided to the control group participants, according to their preferences. The app will be remotely uninstalled at the backend by the administrators after 9 months.

### Measures

#### Overview

All survey questionnaires will be accessed via QR codes and answered online, with all fields set as mandatory to prevent nonresponses. The tools to be used in this study are as follows.

#### Demographic Questionnaire

This self-designed questionnaire has been developed based on the conceptual framework of this study. It includes questions on age, sex, BMI, educational level, occupation, number of hospital stays, complications, age at diagnosis, duration of diabetes, frequency of blood glucose self-monitoring, department responsible for follow-up, number of physician changes, and number of department changes.

#### Primary Outcomes

These include HbA_1c_ levels, percentage with HbA_1c_ <7.0%, and the number of hyperglycemic and hypoglycemic events.

##### HbA1c

HbA_1c_ will be recorded as a percentage; data will be obtained through venous blood analysis.

##### Percentage With HbA1c <7.0%

The percentage of HbA_1c_ readings below 7.0% for each participant will be calculated.

##### Hyperglycemic Events

The number of events with blood glucose >200 mg/dL or “high” on the glucometer or diagnosed ketoacidosis will be calculated for each participant.

##### Hypoglycemic Events

The number of events with blood glucose <60 mg/dL or “low” on glucometer or diagnosed hypoglycemia will be calculated for each participant.

For hyperglycemic and hypoglycemic events, during the data collection period, the blood glucose readings will be downloaded from the blood glucose meters of the participants, and the number of hyperglycemic and hypoglycemic events will be calculated. Considering that changes in blood glucose levels may be tracked only after adopting treatment measures, 2 researchers will perform a manual inspection of the data simultaneously. Only the first recorded reading, among all readings taken within the same measurement period, will be analyzed.

#### Secondary Outcomes

##### Perceived Diabetes Self-Management Scale

The original questionnaire was developed by Wallston et al [43]. It consists of 8 items scored on a 5-point Likert scale ranging from 1 (strongly disagree) to 5 (strongly agree), of which items 1, 2, 6, and 7 are reverse scored. The total score ranges from 8 to 40, with higher scores indicating higher confidence in diabetes self-management. Lin et al [44] translated the scale into Chinese to assess 168 patients with T1D and type 2 diabetes (T2D). The translated questionnaire has content validity, Cronbach α, and test-retest reliability values of 0.75, 0.93, and 0.97, respectively.

##### Self-Care Behavior Assessment Scale

This scale was originally developed in Chinese by Wang [45] to measure self-care behaviors of adolescents with T1D. It comprises 7 dimensions: pharmacological treatment–insulin injections; healthy diet–diet control; blood glucose monitoring–self-monitoring; physical activity and exercise–regular exercise; problem-solving–risk reduction; healthy coping–psychological and social adaptation; and stress adaptation. A total of 39 items are scored on a 5-point Likert scale ranging from 1 (not achieved at all) to 5 (completely achieved), of which items 26 to 35 are reverse scored. The total score ranges from 39 to 195, with higher scores indicating better self-care behaviors. Cronbach α and the content validity value are 0.87 and 0.92, respectively.

##### Diabetes Distress Scale

The original scale was developed by Polonsky et al [46]. The Chinese version consists of 17 items across 4 subscales: emotional burden, physician-related distress, regimen-related distress, and diabetes-related interpersonal distress. Items are scored on a 4-point Likert scale ranging from 1 (no distress) to 4 (severely distressed), with higher scores indicating more severe emotional distress. Cronbach α and test-retest reliability values of the Diabetes Distress Scale are 0.89 and 0.81, respectively [47].

##### Diabetes Quality of Life

The original scale was developed for use in the Diabetes Control and Complications Trial and translated into Chinese by Cheng et al [48]. The Chinese version consists of 42 items with the following subscales: satisfaction, impact, and diabetes-related worry. Each item is scored on a 5-point Likert scale, with 1 and 5 representing “very dissatisfied” and “very satisfied” in the satisfaction subscale, “never” and “always” in the impact subscale, and “never worried” and “always worried” in the diabetes-related worry subscale, respectively. Higher scores are indicative of a higher QoL. The Cronbach α and test-retest reliability values of the scale and subscales are within the ranges of 0.76-0.92 and 0.94-0.99, respectively.

##### Diabetes Knowledge Questionnaire

The original questionnaire was developed by Garcia et al [49]. The Chinese version comprises 24 items that are answered “Yes,” “No,” or “I don’t know”; 1 point is awarded for each correct answer. The total score ranges from 0 to 24 points, with higher scores indicating more excellent knowledge of diabetes. In a study that adopted the 24-item Diabetes Knowledge Questionnaire (DKQ) for assessing 108 patients with T2D, the results indicated reliability and Cronbach α values of 0.78 and 0.89, respectively [50]. Currently, the DKQ is used chiefly for patients with T2D. However, when we sequentially inspected all items of the DKQ to determine its applicability to patients with T1D, it was found that the correct answer for Item 6, “If I am diabetic, my children have a higher chance of being diabetic,” needs to be changed to “No,” and for Item 13, “Medication is more important than diet and exercise to control my diabetes,” needs to be changed to “Yes.” In contrast, the remaining items and answers remain applicable to patients with T1D and are considered essential knowledge among such patients. Therefore, the DKQ will be used in this study following the above-mentioned modifications.

#### App Use Data

During the data collection period, the administrators will also download app-use data at the backend. Such data include log-ins to the Healthcare CEO app, the number of e-books read in the CEO Knowledge Base, the number of online consultations with the CEO’s Secretary, and the content of the chats in the CEO Chat Rooms.

### Data Collection

Three research assistants with a background in health care, who have a basic understanding of T1D, have undergone training, and are not involved in the provision of health care, will be responsible for the data collection. Attending physicians of patients with T1D will provide referrals of patients who meet the inclusion criteria. One research assistant in charge of data collection will then approach the patients to explain the purposes of the study and the research process. Patients who agree to participate will subsequently fill out the informed consent sheet and undergo the preintervention test (T0) in an undisturbed environment. The second research assistant will obtain the randomization results from the statistician by phone and assist participants in downloading the Healthcare CEO app onto their smartphones based on their group assignment. The third research assistant will check and remind the second research assistant of the intervention steps to ensure the accuracy and completeness of the intervention. Considering that HbA_1c_, the critical indicator of blood glucose control, is measured once every 3 months, the time points for the postintervention data collection for both groups are set at 3 months (T1), 6 months (T2), and 9 months (T3) after the intervention.

### Treatment Fidelity

#### Overview

To monitor the faithfulness and fidelity of the intervention process and enhance intervention effectiveness evaluation, we formulated strategies for the inspection of treatment fidelity in the 5 areas proposed by Bellg et al [51] at the National Institution of Health Behavior Change Consortium.

#### Treatment Design

A standard treatment manual was formulated to explain the purpose of the study, the processes, and the details. Standardized interaction content of the interventions was established.

#### Training Provider

Research assistants received 16 hours of standardized training in accordance with the roles they will serve in the study. The standard training sessions attended by all research assistants included treatment of patients with T1D in the transition phase, research ethics, and 2 observational visits to the outpatient clinic for familiarization with the study settings. Individual training sessions included data collection techniques or the development, methods of use, and troubleshooting of the Healthcare CEO app. In addition, during the data collection process for the first 10 participants, the research assistants will be accompanied by senior researchers to confirm their techniques and abilities in participant inclusion and ensure faithfulness in the implementation of the intervention measures, and strategies for improvement will be provided after observation.

#### Delivery of Treatment

An intervention step checklist has been formulated and will be provided to research assistants for the sequential inspection and confirmation of the level of completion and completeness of the intervention measures. Another assistant will inspect the fidelity and faithfulness of the intervention process using an intervention process checklist and perform voice recordings of the entire treatment process for researchers to conduct random analyses, evaluations, and feedback.

#### Receipt of Treatment

Participants will be asked to perform operations in the app and discuss and clarify any doubts that they may have. An intervention content checklist will be provided to the participants after the completion of the intervention to confirm that they have received the complete intervention and determine their level of understanding and app use abilities.

#### Enactment of Treatment Skill

To ensure that participants are capable of using the app in everyday life, the following will be carried out: weekly monitoring of the frequency of app content usage and the level to which app usage has been correctly completed, documenting usage issues and suggestions highlighted by participants during routine telephone follow-up, and downloading and analyzing app use data during each round of data collection.

### Data Analysis

#### Statistical Analysis

The intention-to-treat principle will be used for the data analysis; that is, all participants will be included in their initially assigned groups for analysis, regardless of whether they withdraw from the study for any reason or are lost to follow-up. Data will be analyzed using IBM SPSS 26.0 for Windows, with differences considered statistically significant at *P*<.05. Demographic and disease status data, including age, gender, BMI, age at diagnosis, disease duration, and HbA_1c_, along with metrics on the app usage to evaluate treatment fidelity, such as the log-ins to the app, frequency of use for each interface, number of e-books read in the CEO Knowledge Base, and number of online consultations in CEO’s Secretary, will be presented through descriptive statistics. This will include frequency distributions, percentages, means, SDs, and maximum and minimum scores. Homogeneity in the essential attributes of the 2 groups will be compared and analyzed using inferential statistics, such as the chi-square test and ANOVA. Owing to the longitudinal nature of this study, repeated data measurements will be taken. Dependencies may exist among the data at different time points, and data loss may also occur. Using generalized estimating equation models, repeated measurements can be simultaneously included in the regression analysis. Through the variance correction process, consistent estimates of the regression parameters and their variance can be provided to correct for dependencies, and participants with missing values can still be included in the analysis [52]. Therefore, a generalized estimating equation will be used for data analysis to compare the differences between the experimental and control groups at both preintervention and postintervention time points, as well as the differences in the scores of the experimental group before and after intervention.

#### Healthcare CEO App Chat Room Data Analysis

Chats in the CEO Chat Rooms will first undergo thematic categorization, beginning with a review of the chat content to gain an initial understanding. Relevant patterns will then be identified and labeled through initial coding, and similar codes will be grouped to form broader themes. These themes will be reviewed and refined for relevance and consistency with the data, and each theme will be clearly defined. Finally, the frequency of each identified theme will be calculated.

### Ethical Considerations

The study will follow the Declaration of Helsinki and has received approval from the Ethics Committee of the Chang Gung Foundation Institutional Review Board (submission reference: 202100050B0; March 9, 2021). It will uphold key ethical principles, including informed consent, with participants receiving comprehensive information about the study. For minors, consent will be obtained from a legal guardian simultaneously, and efforts will be made to minimize any undue influence from guardians or family members to guarantee voluntary participation. Participants may withdraw at any time without impacting their medical care. The study prioritizes participant safety through self-reported questionnaires and a mobile app that poses no risks. Justice is maintained by ensuring equal medical care for all participants, with the control group accessing the app after the study. Privacy is protected with data secured in locked storage and coded identifiers used in publications to ensure anonymity. Participants will receive a voucher worth NT $300 (approximately US $9.34) for each completed questionnaire as an appreciation for their time and effort.

### Validity and Reliability

The study design and participant enrollment process were formulated in accordance with the recommendations of the CONSORT (Consolidated Standards of Reporting Trials) statement. We also formulated strategies based on the National Institution of Health Behavior Change Consortium guidelines to monitor the faithfulness and fidelity of the intervention process. The research tools used in this study also exhibit good validity and reliability. In the measurement of the primary outcome variables, this study differs from previous works on diabetes management apps in that “% with HbA_1c_ <7.0%” data will be calculated for the first time for analysis. This allows for greater objectivity and rigor in evaluating the effectiveness of the Healthcare CEO app.

## Results

**Figure 3:**
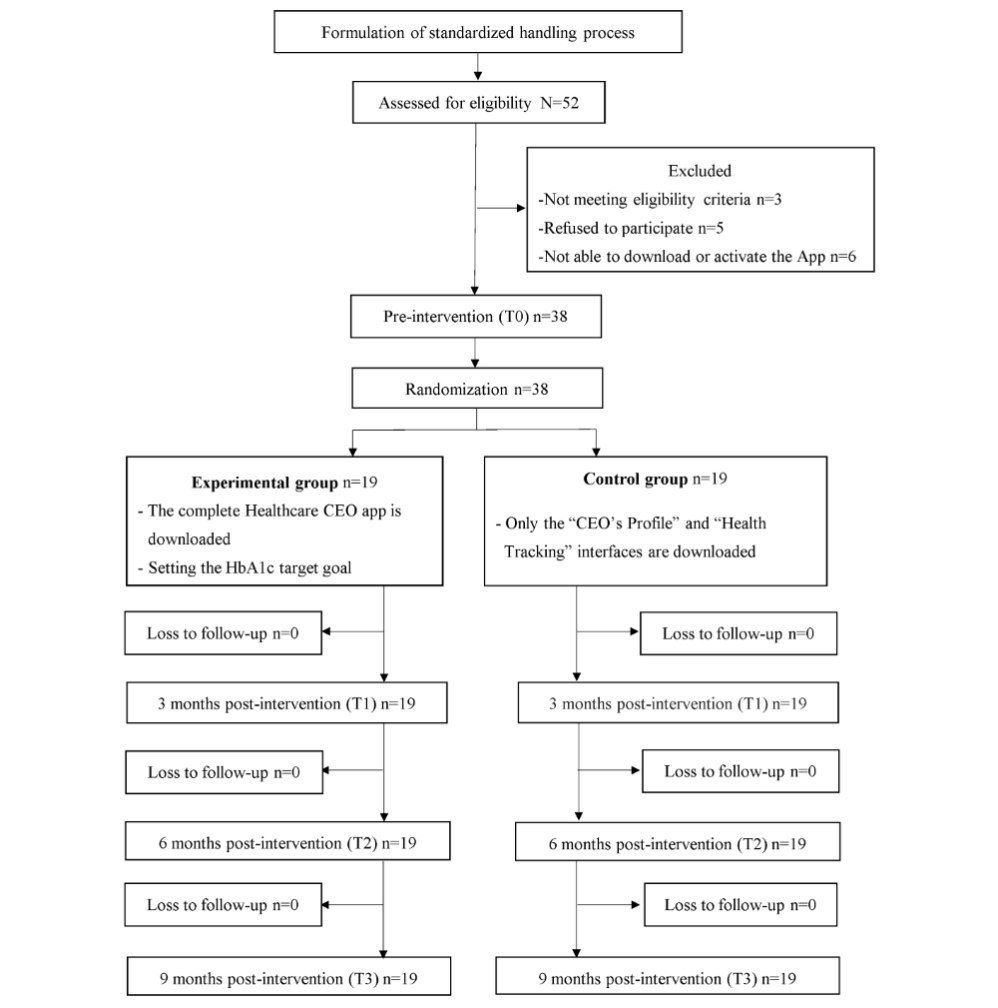
CONSORT (Consolidated Standards of Reporting Trials) flowchart of preliminary results of the trial. HbA_1c_: hemoglobin A_1c_.

After a comprehensive review, the National Science and Technology Council, Republic of China (MOST 109-2314-B-182-057-MY2), and Chang Gung University (NMRPD1K1021) provided financial support for this research project. The project was initially scheduled to run from August 1, 2020, to July 31, 2022. However, owing to the widespread impact of the COVID-19 pandemic, the study was abruptly halted after the enrollment of 38 participants. Details of the recruitment and randomization processes can be found in the CONSORT flowchart (Figure 3). We anticipate that the preliminary results will be compiled and submitted for publication by November 2024. The recruitment of participants resumed in August 2023, with findings expected to be finalized by July 2025. At present, each experimental and control group includes 13 participants.

## Discussion

### Principal Findings

The Healthcare CEO app distinguishes itself as one of the few evidence-based, multifunctional integrated apps specifically designed for individuals aged 16-25 years managing T1D. This study uses a double-blind, randomized controlled trial intervention, with anticipated outcomes aimed at enhancing disease knowledge, improving self-care skills, and strengthening self-efficacy in disease management. Additionally, training in effective communication skills is intended to reduce interpersonal and emotional distress, enhance QoL, and ultimately improve glycemic control outcomes. The strengths and limitations of this study are elaborated in the following sections.

### Strengths

The 6 major aspects of diabetes self-management are physical activity, nutrition, blood glucose testing, medications or insulin injections, health feedback, and education. However, existing diabetes management apps focus only on 2 to 3 of these aspects on average. App developers should work closely with health care providers and patients to ensure that the developed apps address patients’ health care needs [53]. Our app differs from the majority of commercially available apps in that it contains the following distinctive features: (1) the app content has been designed based on a series of empirical studies [5,23,42] to ensure that it fulfills the needs of the users; (2) the app contains diverse content across 11 interfaces instead of being limited to 2 or 3 aspects; (3) the See Here: Diet and Exercise interface allows for easy and convenient recording of carbohydrate and calorie intake and exercise intensity; (4) the CEO Knowledge Base contains specific content targeted at patients in the transition phase, which is divided into basic and advanced knowledge, for perusal on a need basis; (5) themed discussions can be organized in the CEO Chat Rooms interface, during which users can discuss their needs and expert suggestions can be provided; (6) an SOS Calls function is provided for users transitioning from the dependence to independence phases, so that they can seek help from trusted individuals when needed; and (7) a points system, in which points can be exchanged for gift vouchers, is used to reward users for app use. With the above features and added emphasis on enhancing treatment fidelity, we anticipate that our results will indicate high effectiveness in diabetes self-management among patients with T1D in the transition phase due to our app-based intervention.

### Limitations

This study design has several limitations. First, the app usage may be limited by an unstable internet connection, which could impact the effectiveness of our app-based intervention. Second, in addition to providing gift vouchers to enhance the motivation and efforts of the participants, we also factored in an allowance during sample size estimation to minimize the effects of participant withdrawals. Third, because of the nature of the study, the researchers cannot be blinded during the study implementation, which may lead to potential biases. To address this issue, researchers must adhere to strict intervention principles to minimize the impact on the study results. Finally, the Healthcare CEO app was developed and will be evaluated based on Taiwanese participants with T1D in the transition phase. Therefore, caution will have to be exercised, and cultural differences will be considered when interpreting the study results.

### Conclusions

Patients with T1D transitioning from adolescence to early adulthood are in extreme need of support from health care providers and primary caregivers. The Healthcare CEO app developed in this study differs from current commercially available apps in that the functions desired by transitioning adolescent patients, as indicated by the empirical results obtained from previous qualitative and quantitative studies, have been integrated into the app to the best of our ability. In other words, this app is the first multifunctional app targeted at the needs of patients with T1D in the transition phase from adolescence to early adulthood. Our study protocol consisted of strategies formulated to enhance the treatment accuracy of the intervention measures. Such strategies make it possible to increase the objectivity of intervention effectiveness evaluation. The findings can serve as a reference for further applications to the clinical care of patients in the transition phase.

## Abbreviations

CONSORT: Consolidated Standards of Reporting Trials
DKQ: Diabetes Knowledge Questionnaire
HbA1c: hemoglobin A_1c_
QoL: quality of life
T1D: type 1 diabetes
T2D: type 2 diabetes

## Data Availability

The datasets produced or analyzed in this study can be obtained from the corresponding author upon reasonable request.
